# Supplementary material for: Dengue Virus NS1 Binds Ephrin B1 to Trigger Endothelial Dysfunction
Source: bioRxiv. 2025 Nov 19:2025.11.19.689067. Preprint. [Version 1] doi: 10.1101/2025.11.19.689067 (PMC12668011; doi:10.1101/2025.11.19.689067)
Supplement: Supplement 1 [file media-1.pdf]

Supplementary Material

Dengue Virus NS1 Binds Ephrin B1 to Trigger Endothelial Dysfunction

Felix Pahmeier, Sabrina R. Hammond, Charlotte Flory, Xinyi Feng, Saeyoung E. Lee, Erika V. Jimenez-Posada, Elias M. Duarte, Jaime A. Cardona-Ospina, Aquena H. Ball, Nharae E. Lee, Kayla Leung, Laurentia V. Tjang, P. Robert Beatty, Scott B. Biering, Pietro Scaturro, Eva Harris

Figures S1-S4

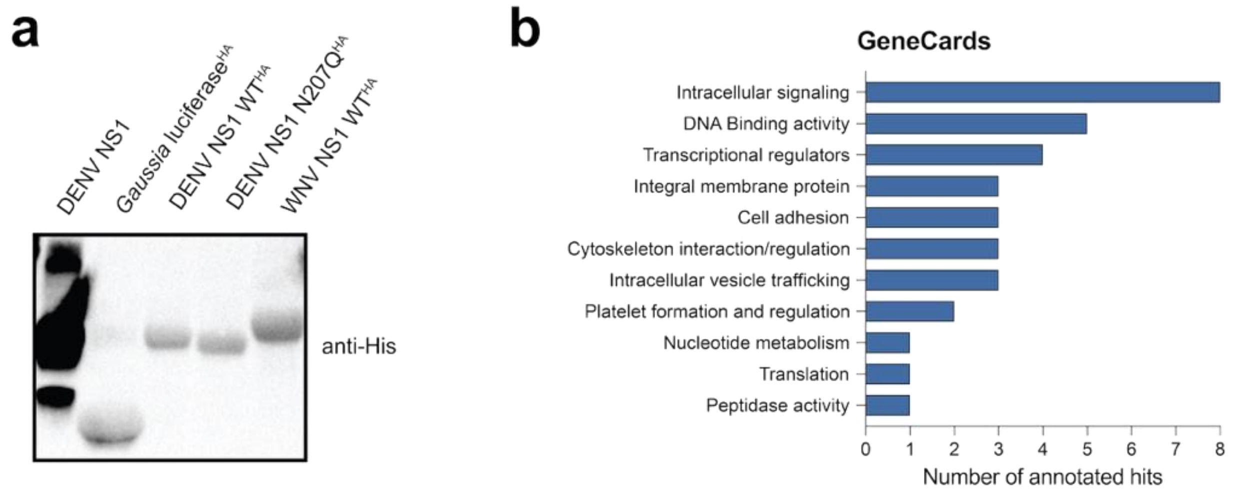

Figure S1. **Recombinant protein validation and GeneCards analysis of host interaction partners**. **a**, Recombinant HA- and His-tagged proteins were analyzed by Western blot using anti-His antibodies. **b,** GeneCard annotations of DENV NS1 WT-selective host factors.

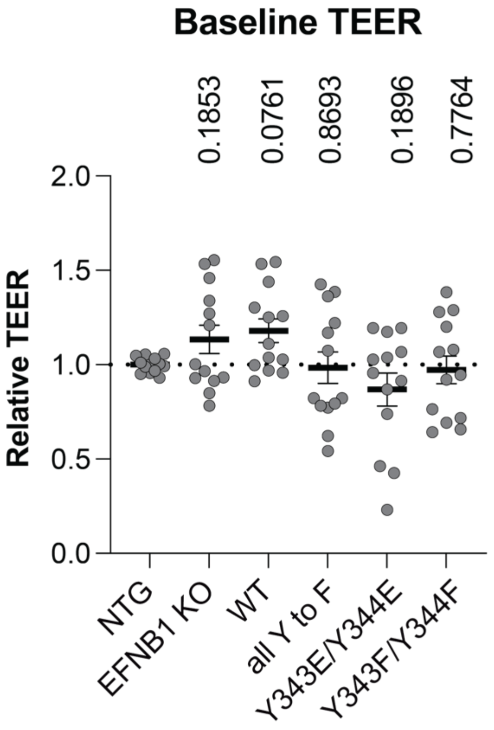

Figure S2. Genetically modified HPMEC cell lines form endothelial barriers *in vitro*. TEER values at 0 and 24 hours post-treatment of the HPMEC EFNB1-KO cell line transduced with EFNB1 expression cassettes. Statistical comparisons were performed by ordinary one-way ANOVA and *P*-values are shown on the top of each dataset. NTG, non-targeting guide; KO, knockout.

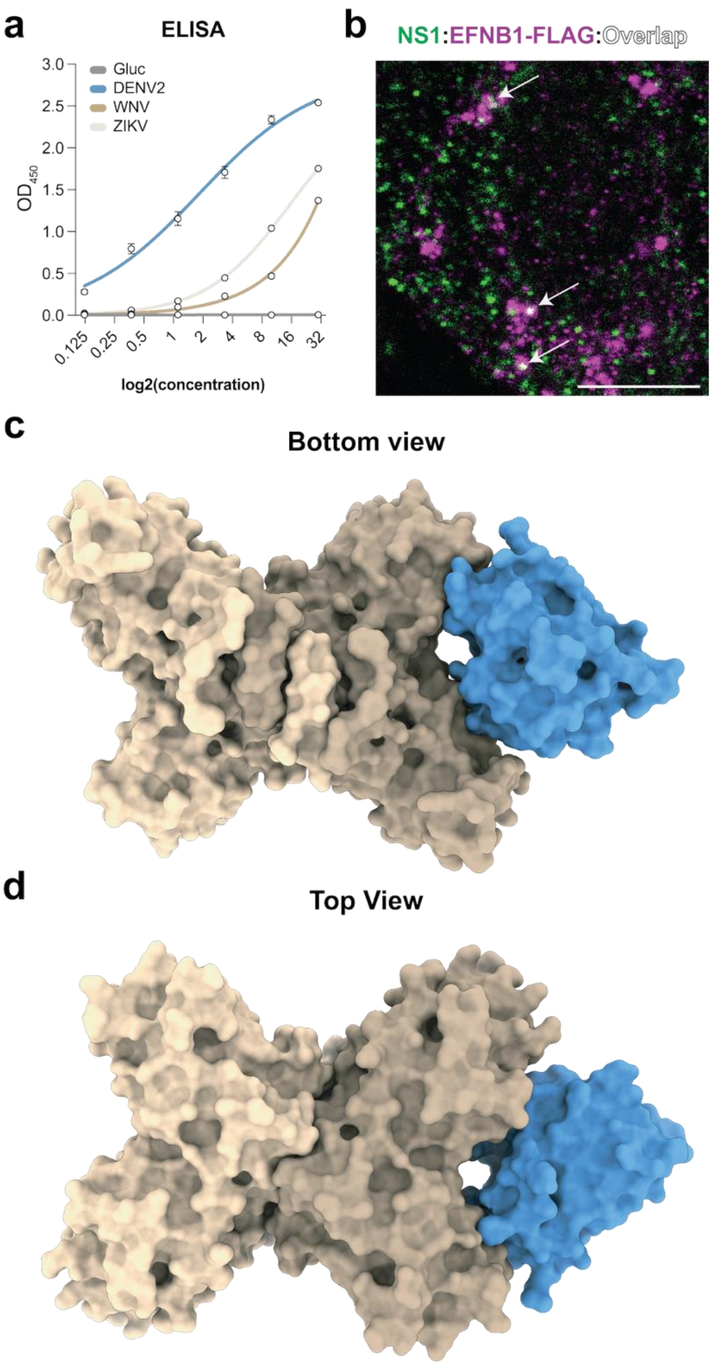

Figure S3. EFNB1 binds and co-localizes with NS1. **a**, Binding of flavivirus NS1 proteins to EFNB1-Fc fusion in an ELISA. Plates were coated with the EFNB1-RBD Fc fusion protein, a dilution series of the indicated His-tagged NS1 proteins was added, and their binding was detected using an anti-His antibody (n=2). **b**, EFNB1-FLAG-expressing cells were treated with NS1 or left untreated and incubated for 30 minutes at 4°C and then fixed. DENV NS1 (green) and EFNB1-FLAG (purple) localization was detected with mAbs targeting the His or FLAG tag, respectively (scale bar, 10 μm). **c-d**, Surface representation of EFNB1 RBD (blue) interacting with dimeric DENV2 NS1 (tan) as predicted by Boltz2.

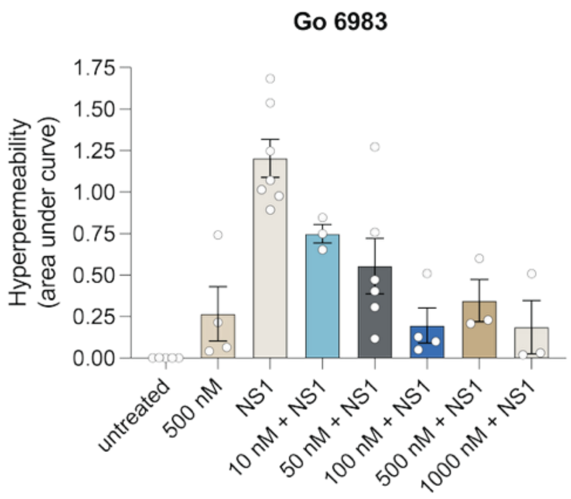

Figure S4. PKC inhibition can block NS1-mediated endothelial dysfunction. HPMECs were treated with NS1 in absence of decreasing concentrations (in nM) of the PKC inhibitor Go 6983, and the electrical resistance was measured at 0, 6 and 24 hours post-treatment. The area under the curve was plotted as the mean ± SEM (n≥2).
